# Minimally invasive 360-degree pelvic ring fixation using a combination of crab-shaped fixation and pelvic internal fixator for unstable pelvic ring fracture: A case report

**DOI:** 10.1016/j.tcr.2021.100540

**Published:** 2021-10-01

**Authors:** Yoshiyuki Kamatani, Akinori Okuda, Naoki Maegawa, Hiroaki Matsumori, Hideki Shigematsu, Kenji Kawamura, Hidetada Fukushima, Yasuhito Tanaka

**Affiliations:** a Department of Emergency and Critical Care Medicine, Nara Medical University, 840 Shijo-cho, Kashihara City, Nara 634-8522, Japan; b Department of Orthopedic Surgery, Nara Medical University, 840 Shijo-cho, Kashihara City, Nara 634-8522, Japan; c Kashiba Asahigaoka Hospital, Department of Orthopedic Surgery, 839 Uenaka, Kashiba City, Nara 639-0265, Japan

**Keywords:** Pelvic ring fixation, Crab-shaped fixation, INFIX, Spinopelvic fixation, Case report

## Abstract

Surgery with both anterior and posterior fixation is recommended for unstable pelvic ring fractures; nonetheless, the surgical method remains controversial. Crab-shaped fixation is a minimally invasive and strong posterior fixation method using spinal instruments that can reduce vertical dislocations. The use of pelvic internal fixator as a minimally invasive anterior fixation method has been reported. It is recommended in cases where there is an open wound in the lower abdomen or damage to the pelvic organs. Conversely, to the best of our knowledge, there has been no report on the combined use of crab-shaped fixation and pelvic internal fixator to date. We performed a minimally invasive 360-degree fixation using a combination of crab-shaped fixation and pelvic internal fixator for an unstable pelvic ring fracture (AO-C2) and sacral fracture (Denis zone II) with 15-mm vertical dislocation. The sacral fracture was accompanied by a large bone fragment in the spinal canal, which was suspected to have caused neuropathy. Therefore, in addition to posterior fixation, we performed decompression and removed the bone fragment. Postoperative computed tomography revealed that the sacral vertical dislocation was reduced to 7.5 mm. The patient started getting out of bed on postoperative day 2. His neuropathy improved after surgery. Owing to abdominal discomfort, pelvic internal fixator was extracted at 3 months postoperatively. Bone fusion was completed, and posterior fixation was removed at 9 months postoperatively. Two years after, the patient walks independently and has returned to work. Minimally invasive 360-degree pelvic ring fixation is a treatment option for an unstable pelvic ring fracture (AO-C2).

## Highlights

• CSF360 is a modified CSF surgical technique.
• Free bone fragment and 360° pelvic ring fixation CSF and INFIX were performed.
• Unstable pelvic ring fractures can be fixed to 360° of circumference.

## Background

To select the appropriate posterior fixation technique for the treatment of unstable pelvic fractures, confirming their stability based on fracture type is necessary [1]. An unstable pelvic ring fracture may cause severe complications or pelvic deformities.

Lumbosacral spinal fixation is recommended because it is robust, minimally invasive, and involves less bleeding [2]. Crab-shaped fixation (CSF), a recently developed lumbosacral spinal fixation technique, uses a spinal instrument to achieve minimal invasiveness [3]. It is useful for fixating unstable pelvic ring fractures of the following types: AO/OTA61C1.3 with large vertical dislocations, AO/OTA61C1 without large vertical dislocations and with Denis zone III, and AO/OTA61C2, C3 with or without large vertical dislocations. Similarly, pelvic internal fixator (INFIX), a minimally invasive anterior fixation device [4], [5], is recommended for open wounds in the anterior region, pelvic organ injury, or when a strong, minimally invasive anterior fixation is specifically required.

Herein, we describe a case of AO/OTA61C2 unstable pelvic ring fracture with large vertical dislocation of the sacrum complicated by protrusion of a giant bone fragment into the spinal canal.

## Case presentation

**Fig. 1:**
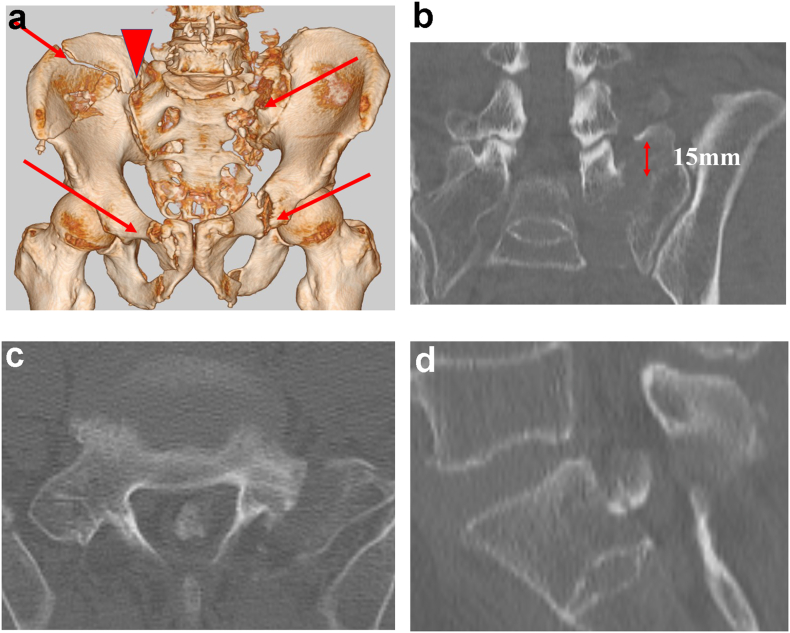
Preoperative computed tomography scan a: Arrows indicate fracture sites; arrowhead shows the sacroiliac joint dissociation. b: Sacral fracture with 15-mm vertical dislocation. c, d: A large free bone fragment in the spinal canal. (c, Axial view; d, sagittal view).

The patient was a 59-year-old man with an unremarkable medical history who worked as a civil engineer. During an accident at work, he got buried deeply under a large amount of sand, collapsed, and then transported to the emergency department of our medical center (day 1). Computed tomography (CT) revealed bilateral pubic and sciatic fractures, anterior partial separation of the right sacroiliac joint, and left sacral fracture (Denis zone II, Fig. 1a) accompanied by a 15-mm vertical dislocation (Fig. 1b). Additionally, a giant bone fragment (25 × 5 mm^2^) was present in the spinal canal at the S1 level (Fig. 1c, d). He had numbness at the left S2 region but no complaints related to motor impairments or bladder and rectal dysfunctions. He was, therefore, diagnosed with S2 nerve root compression caused by the bone fragment in the spinal canal and thus patient required immediate removal of the bone fragment and decompression to improve the numbness of S2 root maximally. He underwent surgery on day 2.

**Fig. 2:**
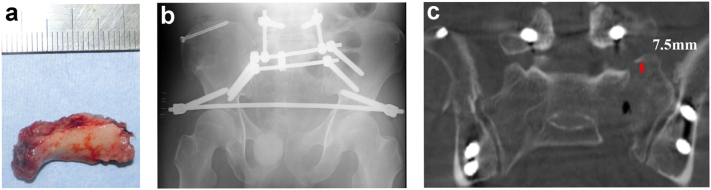
a: Extracted spinal canal fragment that measured 25 mm × 10 mm. b: Postoperative plain radiograph shows the iliac tips and internal fixator screws intersecting at the acetabulum. c: Postoperative computed tomography scan shows vertical reduction from 15 mm to 7.5 mm.

First, a robust minimally invasive anterior fixation was performed by INFIX in the supine position in a time-effective approach to prevent further deterioration of the S2 nerve root compression when changing position. After INFIX, he was moved to a prone position. Then, decompression was performed by laminectomy to release the S2 nerve root, and the large bone fragment was removed (Fig. 2a). Finally, sacral vertical reduction and strong posterior fixation were performed by the CSF method (Fig. 2b).

**Fig. 3:**
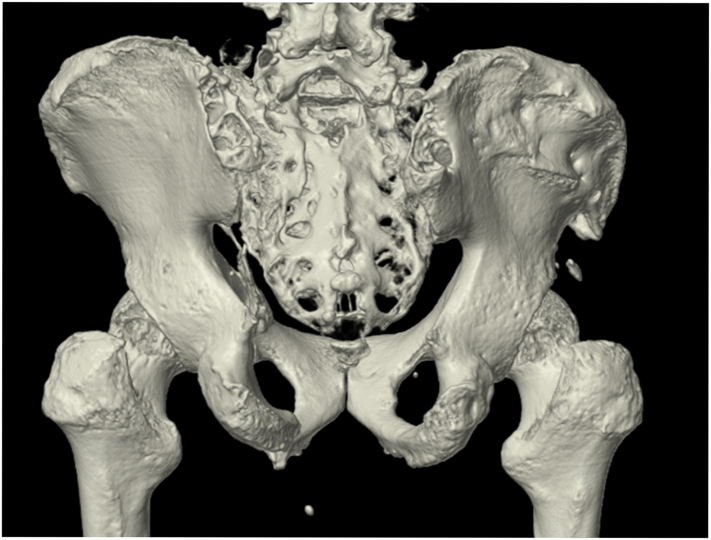
Computed tomography results at 2 years postoperatively showing appreciable bone fusion.

The operative time was 90 min on INFIX and 237 min on laminectomy and CSF. The estimated blood loss was 400 mL. Postoperative CT proved that vertical dislocation of the sacral fracture had reduced to 7.5 mm (Fig. 2c). Sensory numbness was alleviated; however, the patient experienced discomfort and pain due to protrusion of the anterior screw and rod, so the anterior implant was removed 3 months post-surgery. The posterior implant was removed within 9 months after the first operation once bone union development was confirmed. CT performed at 2 years post-surgery clearly showed bone union (Fig. 3). The patient still had mild numbness in the affected leg but could walk independently without a cane and had returned to his original position at work 1 year post-surgery. The Japanese Orthopedic Association score improved to 25/29 from 15/29 at 3 months post-surgery.

The patient provided informed consent for the publication of this report.

## Discussion

Anterior fixation combined with posterior fixation is recommended for the treatment of unstable pelvic ring fractures. Internal fixation is superior to external fixation in terms of biomechanics [6]. Other internal fixation methods have been reported, with the use of branch screw, pubic plate, and INFIX [4]. According to Yin et al., 10 of 35 INFIX patients experienced transient lateral femoral neuropathy and postoperative discomfort due to a subcutaneous rod. However, their symptoms improved after the implants were removed, which explains the need for extraction [7]. Compared with other fixation types, INFIX causes less soft tissue invasion and is associated with fewer reports of wound infections [5]. In our case, pelvic ring fracture of AO/OTA61C2 had high degree of instability and was associated with sacral radiculopathy due to a large bone fragment. Therefore, we considered pelvic ring stabilization and decompression of the left S2 nerve root by removing the large bone fragment. Decompression of the nerve root required a posterior approach in the prone position. Changing the position from supine to prone before pelvic ring fixation could cause deterioration of neurological symptoms when the big bone fragment shifted because of the severe pelvic ring instability AO/OTA61C2. Accordingly, we considered it desirable to stabilize the pelvic ring in the supine position before decompressing the nerve in the prone position. However, nerve root decompression and posterior fixation are time-consuming. Therefore, INFIX, which is minimally invasive and can achieve anterior stability in a time-effective approach, was selected. Because neurological symptoms improved post-surgery, we conclude that INFIX can prevent exacerbation of neurological disorders during positional changes.

Among unstable pelvic ring fractures, sacral fractures with ≥10-mm vertical dislocation cause lumbosacral plexus neuropathy [8]. Therefore, vertical dislocation reduction is necessary. Conventional surgery involves vertical reduction performed by intraoperative traction of the ipsilateral leg. In patients with ipsilateral femoral neck and diaphyseal fractures, lower limb traction cannot be used, and externally fixed half pins and intraoperative reduction pins are unsuitable for the proximal femur. Spinopelvic fixation [2], [9] and CSF [3], which require spinal instruments that can reduce vertical dislocations, have been reported. In these methods, vertical reduction can be performed by applying distraction between the pedicle and iliac screws or by placing a rod connecting both iliac screws. A surgical technique combining INFIX and iliosacral screws provided biomechanical stability against unstable pelvic injury in the vertical and rotational directions [10], suggesting that surgery combining INFIX and CSF is equally stable. In our case, by using CSF, the 15-mm vertical dislocation was reduced to 7.5 mm after INFIX, and good reduction fixation was obtained without any symptoms of lumbosacral plexus post-surgery. Generally, vertical dislocation reduction should be performed before fixation. However, we performed pelvic ring stabilization by INFIX before vertical dislocation reduction. Therefore, we feared that reduction was impossible in the prone position. Despite this, we considered that the strong vertical reduction force of CSF and its spinal instruments could allow sacral vertical dislocation reduction because INFIX uses anterior fixation with one screw for each of the left and right ilia, and the absolute stability of both iliac bones had not been obtained. We could reduce the sacrum to a vertical dislocation of <10 mm using CSF after INFIX. At 9 months postoperatively, bone union was obtained, and the implant was removed. This case demonstrated that the pelvic ring can be fixed to 360° of circumference by combining CSF and INFIX. Hence, we named this CSF technique as 360-degree CSF (CSF360). As the fixation strength was similar to that in previous reports, we ascertained that desired bone union had occurred. However, further reports of cases involving CSF360 are necessary.

In conclusion, we described a case of an unstable pelvic ring fracture (AO-C2) successfully treated using a modified CSF surgical technique—CSF360. Our report demonstrates that unstable pelvic ring fractures can be fixed to 360° of circumference.

## Ethics approval and consent to participate

Not applicable.

## Consent for publication

The authors have obtained oral informed consent from patients for the print and electronic publication of this case report.

## Availability of data and materials

The datasets used and/or analyzed during the current study are available from the corresponding author on reasonable request.

## Funding

Not applicable.

## Authors' contributions

YK wrote the initial draft of this manuscript and subsequent revisions. AO is the senior author who is treating the patient; he is responsible for the oversight of the report and editing of the manuscript. All authors have read and approved the final manuscript.

## Declaration of competing interest

None.
